# Attenuated SARS-CoV-2 variants with deletions at the S1/S2 junction

**DOI:** 10.1080/22221751.2020.1756700

**Published:** 2020-05-04

**Authors:** Siu-Ying Lau, Pui Wang, Bobo Wing-Yee Mok, Anna Jinxia Zhang, Hin Chu, Andrew Chak-Yiu Lee, Shaofeng Deng, Pin Chen, Kwok-Hung Chan, Wenjun Song, Zhiwei Chen, Kelvin Kai-Wang To, Jasper Fuk-Woo Chan, Kwok-Yung Yuen, Honglin Chen

**Affiliations:** a Department of Microbiology and State Key Laboratory for Emerging Infectious Diseases, Li Ka Shing Faculty of Medicine, The University of Hong Kong, Hong Kong SAR, People’s Republic of China; b State Key Laboratory of Respiratory Disease, Institute of Integration of Traditional and Western Medicine, The First Affiliated Hospital of Guangzhou Medical University, Guangzhou Medical University, Guangzhou, People’s Republic of China

**Keywords:** Coronavirus, COVID-19, SARS-CoV-2, Spike mutant, Spike S1/S2 mutant

## Abstract

The emergence of SARS-CoV-2 has led to the current global coronavirus pandemic and more than one million infections since December 2019. The exact origin of SARS-CoV-2 remains elusive, but the presence of a distinct motif in the S1/S2 junction region suggests the possible acquisition of cleavage site(s) in the spike protein that promoted cross-species transmission. Through plaque purification of Vero-E6 cultured SARS-CoV-2, we found a series of variants which contain 15-30-bp deletions (Del-mut) or point mutations respectively at the S1/S2 junction. Examination of the original clinical specimen from which the isolate was derived, and 26 additional SARS-CoV-2 positive clinical specimens, failed to detect these variants. Infection of hamsters shows that one of the variants (Del-mut-1) which carries deletion of 10 amino acids (30bp) does not cause the body weight loss or more severe pathological changes in the lungs that is associated with wild type virus infection. We suggest that the unique cleavage motif promoting SARS-CoV-2 infection in humans may be under strong selective pressure, given that replication in permissive Vero-E6 cells leads to the loss of this adaptive function. It would be important to screen the prevalence of these variants in asymptomatic infected cases. The potential of the Del-mut variants as an attenuated vaccine or laboratory tool should be evaluated.

## Introduction

The outbreak of SARS-CoV2, which emerged in Wuhan, China, in December 2019, has led to the first documented coronavirus pandemic in human history, as declared by the World Health Organization (WHO) on March 11, 2020 [1]. Up to April 10, 2020, 1,600,427 laboratory-confirmed cases have been reported, with an overall mortality rate of 5.98% in 184 countries worldwide. Epidemiological analysis of patients early in the epidemic indicates a probable zoonotic origin of SARS-CoV-2 [2–4]. Genetic characterization and phylogenetic analyses suggest a potential genetic linkage between SARS-CoV-2 and coronaviruses identified in bats in Yunnan, China [5,6]. However, no direct ancestral virus or intermediate host for the cross-species transmission of SARS-CoV-2 to humans has yet been identified. Notably, efficient human to human transmission appears to have occurred as soon as the outbreak of SARS-CoV-2 was recognized [7], suggesting the virus may have crossed to humans for a period of time before it was detected. Study of viral shedding dynamics and host response during the early phase of disease onset suggests SARS-CoV-2 possesses unique ability in human transmission [8]. Human angiotensin-converting enzyme 2 (ACE2) has been identified as the receptor for the spike protein of SARS-CoV-2 and virus entry is also facilitated by the serine protease TMPRSS2 [5,9-11]. By sequence comparison, we found that the spike protein of SARS-CoV-2 is closely related to that of a bat coronavirus, RaTG13 [6], while other parts of the viral genome are more distantly linked [7]. Interestingly, an additional polybasic cleavage motif, PRRA, is found in the S1/S2 cleavage site of SARS-CoV-2 spike protein, but not in the bat coronavirus strain, RaTG13 [12]. Previous studies have shown that protease-mediated entry is one of the determinants of success in SARS coronavirus infection [13,14]. It is speculated that the acquisition of mutations or additional motifs to facilitate S1/S2 cleavage may enhance SARS-CoV-2 infection in humans.

This study analysed purified viral colonies of Vero-E6-cultured SARS-CoV-2 to verify if quasi-species may exist in clinical isolates. We identified a panel of variants (Del-mut) which contains 15–30-bp in-frame deletions in the S1/S2 cleavage site region respectively. However, screening of the original sample and limited numbers of clinical specimens failed to detect these variants. Infection of hamsters revealed that one of the deleted variants (Del-mut-1) is attenuated in its ability to cause disease in this SARS-CoV-2 animal model.

## Materials and methods

### Virus and cells

The isolate of SARS-CoV-2 used in this study has been described in a recent study and was passaged in Vero-E6 cells [15]. Adenocarcinoma human alveolar basal epithelial cells (A549), African green monkey kidney cell, clone E6 (Vero-E6), human epithelial colorectal adenocarcinoma cells (Caco-2) and human lung epithelial cancer cells (Calu-3) were purchased from ATCC and maintained in DMEM supplemented with 10% foetal calf serum (FBS) and 5% penicillin–streptomycin. For virus infection, all cells were infected in with viruses (WT or Del-mut) at 0.01 MOI and infected cells were maintained in DMEM with 5% FBS, plus penicillin–streptomycin.

### Plaque purification

Briefly, confluent Vero-E6 cells were incubated with 10-fold serial dilutions of virus at 37°C for 1 h. The cells were washed and overlaid with 1% agarose gel in virus culture medium. After 2 days of incubation at 37°C, different sizes of plaques were picked using sterile pipette tips and used to infect Vero-E6 cells seeded in 24-well plates, which were then incubated at 37°C for 3 days. Each plaque culture was collected and centrifuged to remove cell debris (2000 rpm for 5 min). The RNA from each cultured plaque was then extracted according to the manufacturer’s instructions (QIAmp viral RNA mini kit, Qiagen). A set of specific primers for detecting the SARS-CoV-2 receptor-binding domain (see below) was used to confirm the identity of each plaque culture. Confirmed plaque cultures were then subjected to an additional round of plaque purification. Full genome sequencing of selected plaque cultures was performed using Sanger sequencing, as described previously [16].

### Quantitative RT–PCR and indirect immunofluorescent assay

Viral RNA was extracted from clinical specimens and virus cultures using a viral extraction kit (Qiagen) and cDNA synthesized using a high capacity cDNA reverse transcription kit (Invitrogen) and random primers, in accordance with the manufacturer’s protocol. WT and Del-mut variant S genes of cultured viruses were amplified by PCR using Taq polymerase (Takara) and the spike RBD-specific primers nCOV-S-F1: 5′-CCACAGACACTTGAGATTC3′ and nCOV-S-R1: 5′-GCAACTGAATTTTCTGCACCA-3′, then gel purified and cloned into the pMD19-T vector (Takara). The plasmids were sequenced and used as standards for qPCR. To detect and distinguish WT and Del-mut variants in clinical specimens and virus cultures, the specific forward primer nCOV-S-F2: 5′-CGTGTTTATTCTACAGGTTCTAATG-3′ was used with the WT specific reverse primer nCOV-S-R-WT: 5′-GCTACA CTAC GTGC CCG CC G AGG-3′ or the deletion mutant specific reverse primer nCOV-S-R-MT 5′-ATGAT GG ATTGACTAGTCTG-3′, respectively. The specificity of qPCR was verified by using WT and Del-mut cDNA as controls. Quantitative PCR was performed using SYBR Premix Ex Taq (Takara) reagent in an LC480 PCR machine (Roche). PCR conditions were as follows: initial denaturation: 95°C for 5 min, 45 cycles of amplification: 95°C for 10s, 60°C for 10 s, 72°C for 10 s, and melting curve analysis: 65°C to 97°C at 0.1°C/s.

For analysis of SARS-CoV-2-N protein in tracheal and lung tissues, immunofluorescence staining was performed on deparaffinised and rehydrated tissue sections using rabbit anti-SARS-CoV nucleocapsid (N) protein antibody together with FITC-conjugated donkey anti-rabbit IgG (Jackson ImmunoResearch, PA, USA), as described in the previous study [15].

### Hamster infection experiment

Eight 7-8-week-old golden Syrian hamsters were divided into 2 groups (WT group: 2 female, 2 male, Del-mut-1 group: 3 females, 1 male). The hamsters were anaesthetized intraperitoneally with ketamine and xylazine and then challenged intranasally with 1.5 × 10^5^ pfu of either SARS-CoV-2 WT or Del-mut virus diluted in PBS. Bodyweight and disease symptoms were monitored daily. On days 2 and 4, lung, nasal turbinate and tracheal tissues were collected from two (day 2 and day 4) hamsters from each group to analyse histopathological changes, assess virus replication by indirect immunofluorescence assay, determine virus titres by plaque assay using Vero-E6 cells, and to screen viruses for changes, including restoration of the 30-bp deletion. All experiments involving SARS-CoV-2 viruses were conducted in a biosafety level 3 laboratory. All animal studies were approved by the Committee on the Use of Live Animals in Teaching and Research, the University of Hong Kong.

## Results

**Figure 1.**
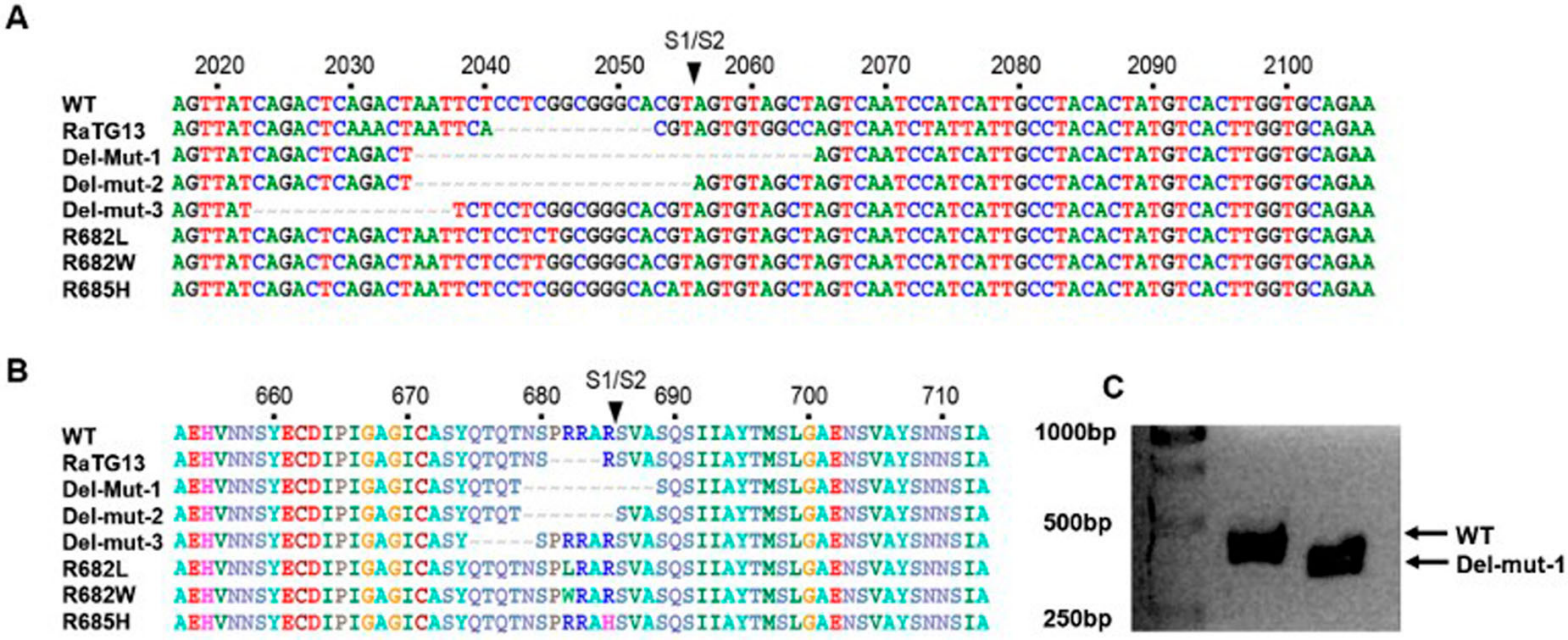
Illustration of nucleotide (A) and amino acid (B) sequence alignments of the S1/S2 junction regions of wild type SARS-CoV-2 (Wuhan-1), 30-bp deleted mutant (Del-mut-1) (EPI_ISL_417443) and the bat coronavirus, RaTG13 [5]. (C) Sizes of WT and Del-mut-1 PCR product on agarose gel amplified with the primers described in the Materials and Methods section.

**Figure 2.**
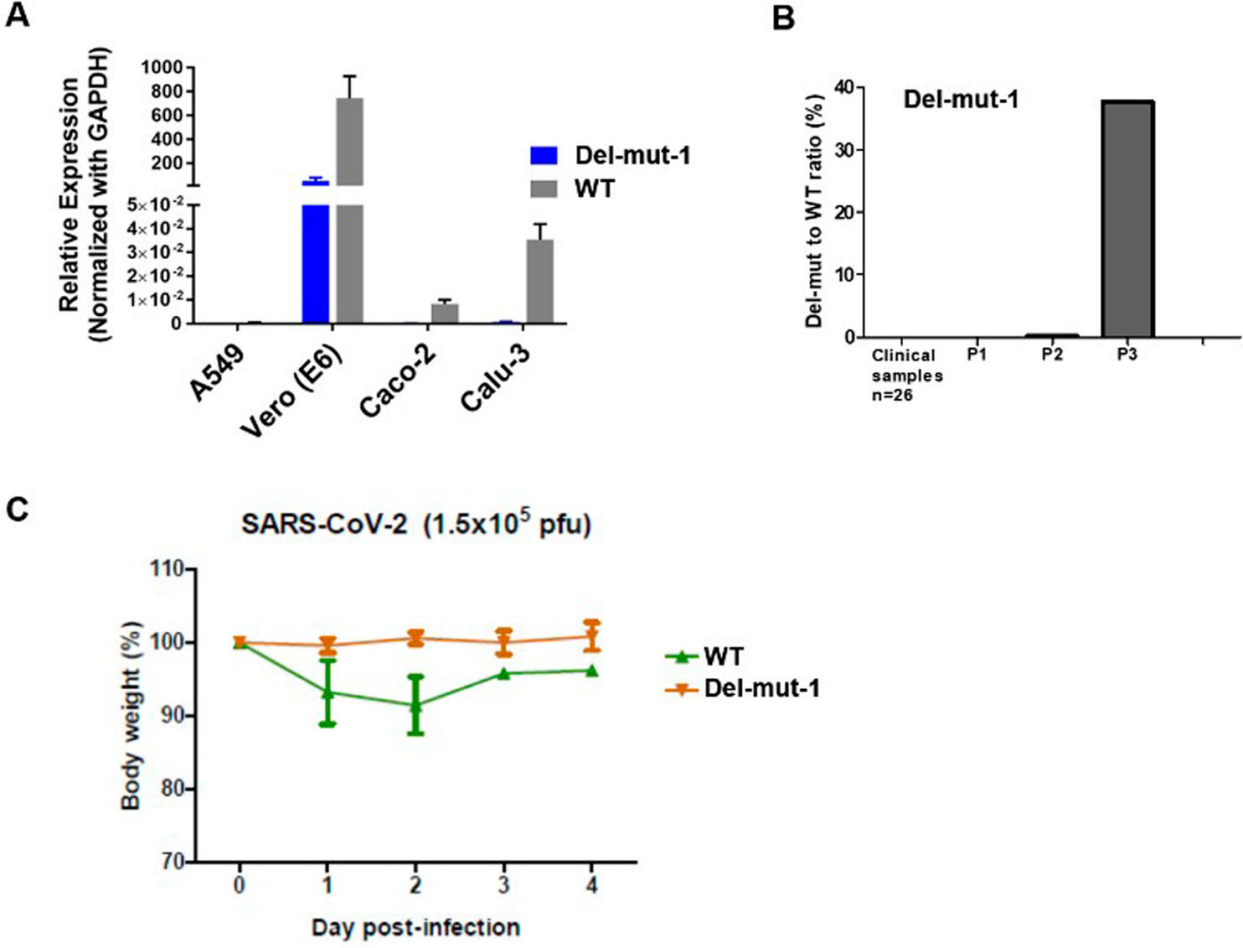
(A) Growth properties of Del-mut-1 and WT viruses in Vero-E6, A549, Caco-2 and Calu-3 cells. (B) Detection of Del-mut-1 variant in clinical specimens (*n* = 26) and different passages of Del-mut-1 variant in Vero-E6 cells. (C) Pathogenicity of WT and Del-mut-1 in hamsters. Bodyweight changes of hamsters (four in each group) infected with WT or Del-mut-1 strains of SARS-CoV-2 virus. Two animals from each group were euthanized on day 2 post-infection (dpi) for the examination of histopathology and virus titers in respiratory tract tissues, and the remainder on day 4 post-infection. Error bars represent mean ± s.d. (*n* = 4 per group for days 0–2, *n* = 2 per group for days 3–4).

Isolation of SARS-CoV-2 from clinical specimens through cell culture may be susceptible to contamination with other respiratory pathogens. To ensure the purity of SARS-CoV-2 for subsequent studies, such as animal infection, we used the plaque purification procedure to purify a clinical isolate. The isolate was initially passaged two times in Vero-E6 cells to prepare a virus stock, with virus from each passage being subjected to plaque purification. From the second passage of the clinical isolate of SARS-CoV-2 [7], we observed different sizes of plaques in cultures. Single-plaque colonies were selected and subjected to the second round of plaque purification. RT–PCR with spike receptor-binding domain (RBD) specific primers confirmed the identity of SARS-CoV-2 after each of the two rounds of purification. Whole-genome sequences of isolates derived from both large and small-sized plaques were analysed. Interestingly, we found multiple variants containing in-frame deletions from 15 to 30 nucleotides and point mutations in the S1/S2 junction. One of the variants contains an in-frame 30-bp deletion which spanned genome coordinates 2035–2064 of the spike region in all small plaque-forming viruses sequenced (Del-mut-1), while no deletion was found in isolates that formed large plaques (WT). Besides this 30-bp deletion, no other deletion or mutation was found in Del-mut-1. Sequence alignment of amino acids shows that this 30-bp deletion is located at the S1/S2 junction (Figure 1). Notably, this deletion removes the PRRA motif from the S1/S2 junction region of the spike protein. To ascertain whether this variant was present in the original specimen or other clinical specimens, we used a pair of primers for specific detection of the Del-mut variant by qRT-PCR (see Materials and Methods for detailed information of primers). However, we could not detect this variant in the original clinical specimen from which this isolate was cultured, nor from another 26 clinical specimens confirmed to be positive for SARS-CoV-2 (Figure 2B). Interestingly, deletion of these 30 base pairs appears to enhance Del-mut-1 variant growth in the mixed population and promote stability through multiple passages in Vero-E6, but not in other human cell lines (Figure 2(A,B)).

**Figure 3.**
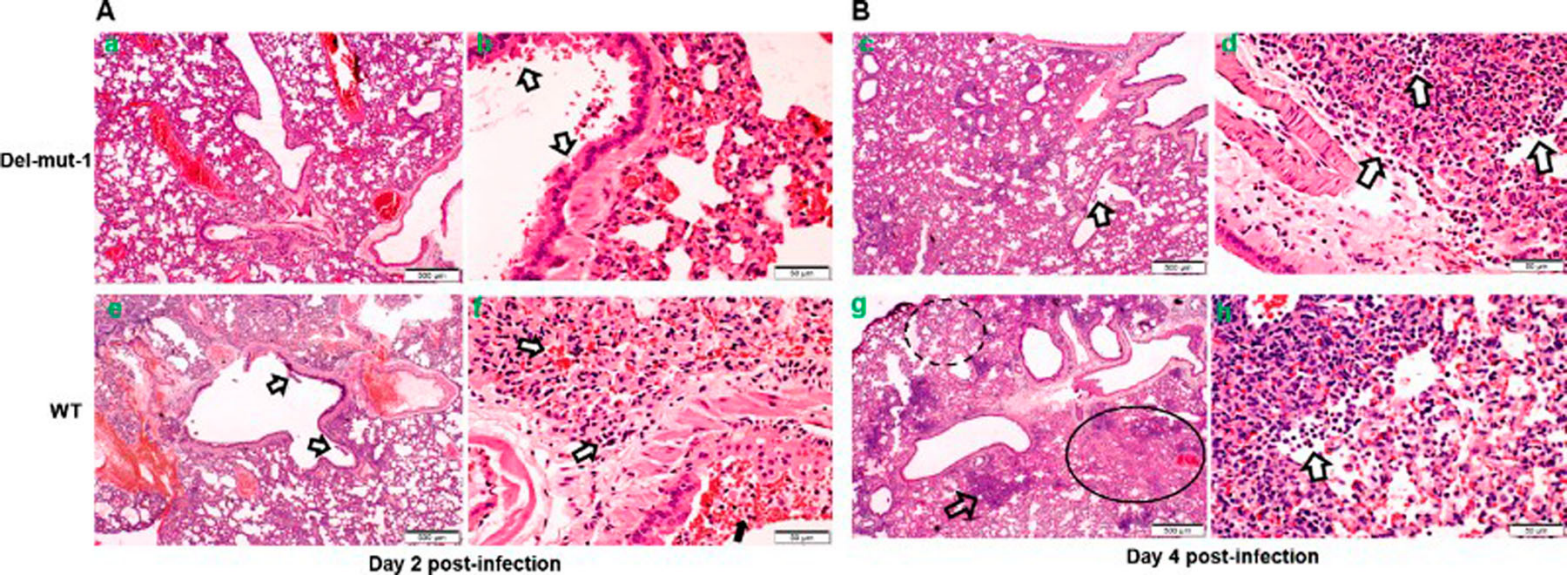
Histopathological analysis of lung tissues from hamsters infected with either WT or Del-mut SARS-CoV-2 virus. Lung tissues were collected on days 2 (A: a, b, e, f) and 4 (B: c, d, g, h) post-infection and processed for HE staining. Images a & b (Del-mut-1, 2 dpi) are representative images showing diffuse alveolar wall thickening, perivascular oedema, and blood vessel and alveolar-capillary congestion involving about 70% of the lung section cutting area. Bronchiolar epithelial cell swelling and a mild degree of desquamation are indicated by arrows in panel b. No infiltration or exudate was observed in the alveolar space. Image c (Del-mut virus, 4 dpi) shows diffuse alveolar wall thickening and peribronchiolar infiltration. The bronchiolar epithelial layer also shows a mild degree of desquamation (arrow). Image d shows detached epithelial cells mixed with mononuclear cells and red blood cells in the lumen of bronchioles. Image e (WT virus, 2 dpi) shows diffuse alveolar wall thickening and peribronchiolar infiltration, with a mild degree of desquamation of the bronchiolar epithelial layer (arrow). Image f shows detached epithelial cells mixed with mononuclear cells, red blood cells in the lumen of bronchiole (solid arrow), alveolar space haemorrhage and mild exudate and cell infiltration. These changes involved about 70% of the lung section cutting area. Image g (WT virus, 4 dpi) shows diffuse alveolar collapse (solid circle) and proline-rich exudates (dashed circle) and a focal area of lung consolidation (arrow). Image h (WT virus, 4 dpi) shows alveolar wall destruction, alveolar space haemorrhage and mononuclear cell infiltration (arrow). Scale bar: 500um (images a, c, e, g), 50um (images b, d, f, h).

**Figure 4.**
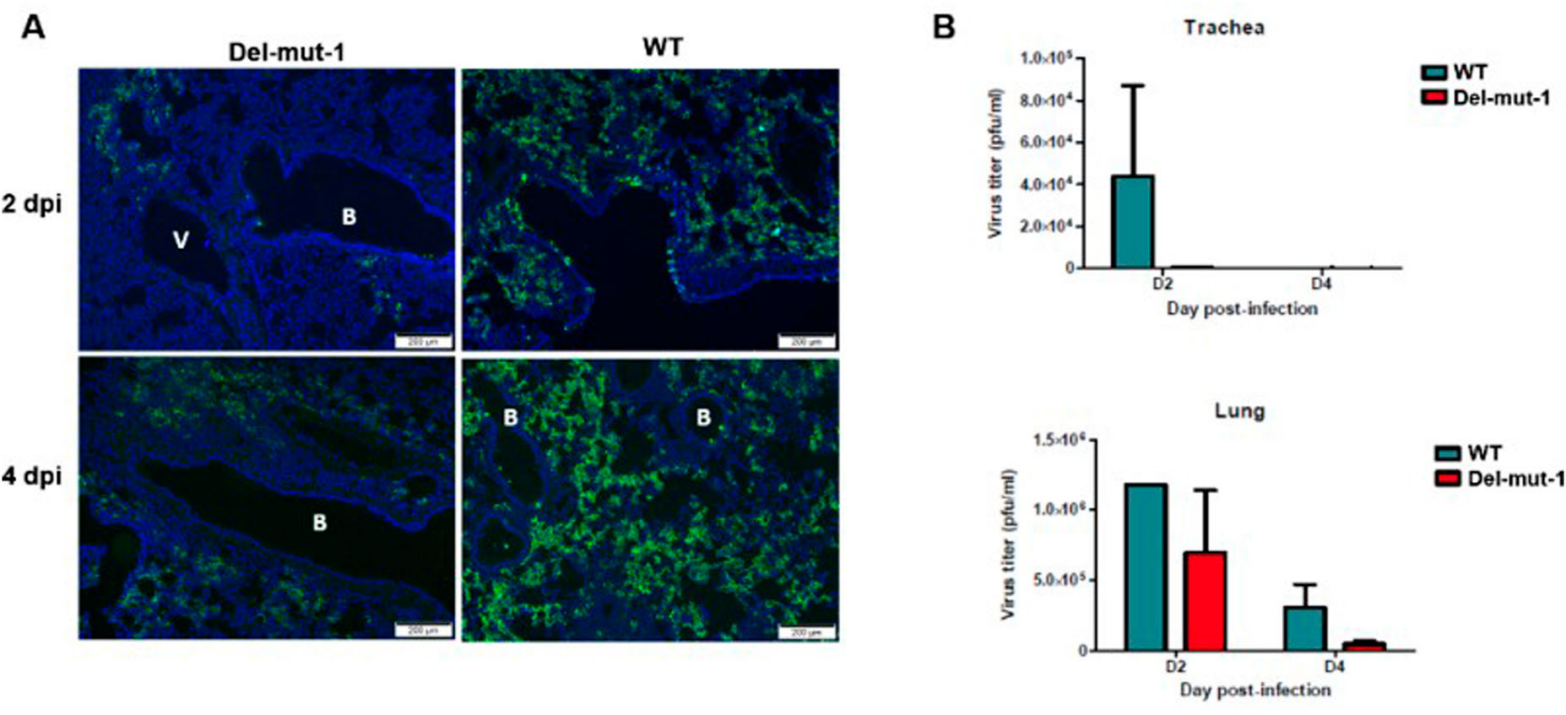
Virus replication in the lung tissues of hamsters infected with either WT or Del-mut-1 SARS-CoV-2 virus. (A) Indirect immunofluorescence assay with antibodies against the N protein of SARS-CoV-2 virus (green). Blue: DAPI staining of cell nuclei. B: bronchiole, V: blood vessel. (B) Virus titration by plaque assay of lung and tracheal tissues collected on day 2 and 4 post-infection. Error bars represent mean ± s.d. (n=2 per group for each timepoint) Scale bar, 200 µm, dpi: days post-infection.

One of the unique features of SARS-CoV-2 is the polybasic cleavage site at the junction of the S1 and S2 subunits of the spike protein. It is possible that deletion of 30-bp (10-aa) in the Del-mut-1 or other variants at the S1/S2 junction may attenuate virus pathogenicity. To test this possibility, we used a hamster infection model which has been shown to be susceptible to SARS-CoV-2 and simulates the clinical and pathological manifestations of coronavirus disease (COVID-19) [15]. Due to the limited number of animals available, only Del-mut-1 was tested in this study. We challenged four hamsters in each group with WT SARS-CoV-2 or the Del-mut-1 strain (1.5 × 10^5^ pfu). Hamsters infected with WT virus began to lose body weight starting from day 1 post-infection, but this seems to stabilize from day 3 post-infection (Figure 2C). In contrast, no apparent body weight loss was observed in hamsters infected with the Del-mut strain. The histopathological analysis shows that while both WT and Del-mut-1 viruses cause infection in hamsters, the WT virus causes more extensive alveolar wall destruction, alveolar space haemorrhage and mononuclear cell infiltration in the lungs of infected animals (Figure 3). Examination of virus titres in the tracheal and lung tissues confirm that wild type virus replicates more efficiently than the Del-mut-1 variant in infected hamsters (Figure 4).

## Discussion

Rapid dissemination of SARS-CoV-2 has led to the first documented coronavirus pandemic [1]. While several coronaviruses, including 229E, OC43, HKU1 and NL63, cause the common cold in humans, available clinical data based on all laboratory-confirmed cases shows that SARS-CoV-2 may cause 1-4% mortality, but this is dependent on age group and geographical locations which may have different availabilities of clinical care [2,3]. It is essential to understand the pathogenic and host adaptative elements of SARS-CoV-2, and since the COVID-19 pandemic is still in the expansion phase, it is also important to know if and when the virus genome stabilizes. We observed a series of deleted mutants (Del-muts) and point mutation variants (Figure 1) in Vero-E6 cell cultures of a clinical isolate. It is possible that the Del-mut variants identified in this study were generated during virus passage in Vero-E6, which is deficient in interferon production, or these variants may be present in very low level among some infected people. Screening more clinical samples for the presence of these variants is necessary. While the original and intermediate hosts have not been identified, SARS-CoV-2 is of zoonotic origin with cross-species transmission resulting in the initial human cases. The emergence of attenuated Del-mut variants in Vero-E6 cells suggests that in permissive conditions the host-adaptive function(s) associated with the junction of S1 and S2 that promote replication in humans were not required, leading to the deletion of variable length of motif (5-10-aa or 15-30-bp) from the region. Of course, it still possible that that the Deletion-mutants and other variants exist in humans as well, since only very limited numbers of clinical specimens have been screened in this study. It is also possible the level of mutant virus in clinical specimens was too low to be detected by our method. As many infections are reported to be mild or even asymptomatic and are not recognized, it would be extremely important to screen for the presence of different variants circulating in people with different degrees of symptoms, including asymptomatic subjects. Our data shows that one of the deletion variants (Del-mut-1) is attenuated in the hamster infection model, causing no bodyweight loss, much milder disease and displaying lower virus replication levels in the lungs. Notably, the examination of Del-mut-1 variant virus isolated from infected hamsters did not detect restoration of the deleted 30-bp sequence. Continued passage of Del-mut-1 variant indicates that the mutant virus is stable and increasing population along the passages in Vero-E6 cells, supporting the contention that the acquired function of the S1/S2 junction insertion is only needed for non-permissive replication.

It is possible that multiple host adaptative strategies are required for the SARS-CoV-2 to be transmitted efficiently in humans. TMPRSS2 protease has been shown to play a critical role in the entry of SARS-CoV-2, and other coronavirus [11,17]. It remains to be investigated if the exceptional transmissibility of SARS-CoV-2 is caused by the acquired function of the S1/S2 junction insertion [18]. It is tempting to suggest that additional protease cleavage motif for gaining virus entry may not be needed in an environment, such as interferon-deficient Vero-E6 cells, where the virus is not heavily restricted. Currently, circulating human coronaviruses associated with the common cold do not contain a basic motif in the S1/S2 junction, so if SARS-CoV-2 is to eventually become a fully human virus, will the one of those Del-mut variants be the final form of SARS-CoV-2 when it is completely adapted to humans? Given its inability to cause severe disease in an animal model, and dependent on safety testing, this attenuated Del-mut-1 variant may prove to be useful for conducting drug screening research or functional studies on SARS-CoV-2 in laboratories where a high-level biosafety facility is not available. One of the most pressing needs in response to COVID-19 is the development of an effective vaccine to contain the ongoing pandemic and in preparation for long term prevalence of the SARS-CoV-2 virus. The safety and vaccine potential of the Del-mut variants should also be explored.
